# Research on Mechanical Behavior of the Steel–Concrete–Steel Composite Structures Subjected to High Temperature of Fire

**DOI:** 10.3390/ma15144872

**Published:** 2022-07-13

**Authors:** Peng Cao, Xuebing Hu, Enlong Liu, Jianzhong Chen, Shouchao Jiang, Hao Ding

**Affiliations:** 1 College of Water Resource & Hydropower, Sichuan University, Chengdu 610065, China; caopeng1@cmhk.com (P.C.); liuenlong@scu.edu.cn (E.L.); 2 National Engineering Research Center of Road Tunnel, Ltd., Chongqing 400067, China; huxuebing@cmhk.com (X.H.); chenjianzhong@cmhk.com (J.C.); 3 China Merchants Chongqing Communications Technology Research & Design Institute Co., Ltd., Chongqing 400067, China; 4 College of Civil Engineering, Tongji University, Shanghai 200092, China; scjiang@tongji.edu.cn

**Keywords:** tunnel engineering, mechanical behavior under fire, model test, the steel–concrete–steel composite structures, temperature distribution, failure mode

## Abstract

A new type of steel–concrete–steel composite structure was adopted and widely used in the immersed tunnel of the Shenzhen–Zhongshan access. The research on the mechanical behavior of the new composite structure under a high temperature of fire is of great engineering significance to the fire protection design of the structure. Both the model test and a numerical simulation were adopted to study the mechanical behavior and damage characteristics of the new composite structure under fire. The RABT standard temperature rise curve was used to simulate the temperature rising law under fire (it reflects the characteristics of temperature rise in case of fire in an enclosed environment: rapidly raised to 1200 °C within 5 min, maintained at 1200 °C for 120 min, then it is cooled to normal temperature after 110 min). The temperature distribution law inside the structure, the deformation development law of the roof and the crack distribution were analyzed based on the thermal–mechanical coupling analysis method. The results showed that the internal part of the composite structure close to the fire surface was directly affected by the high temperature, and the temperature presented a step distribution law, while the part far from the fire surface was affected by the lag effect of the temperature transfer, and the temperature presented a continuous growth law. The roof deformation presented a three-stage model of “rapid growth-deformation stability-deformation recovery” with time. The overall cracks of the composite structure showed a symmetrical distribution under fire. The composite structure presented a shear failure mode as a whole. The results could provide a reference for the study of fire resistance for the new composite structure and support the structural fire protection design of the immersed tunnel of the Shenzhen–Zhongshan access.

## 1. Introduction

China has now become the country with the largest scale, the largest number, the most challenging geological circumstance and structure forms, and the quickest development pace of highway tunnels in the world, thanks to the rapid development of China’s economic construction [1,2,3]. China has a heavily populated river system, multiple straits and bays, and an 18,000 km coastline. Along the river and the sea, about 20 significant cities have been created. China constructed 245 undersea tunnels by the end of 2020, with 23 of them being immersed tunnels, accounting for approximately 10% of the total [4,5,6]). One of the most prevalent disasters in traffic tunnels is fire. The primary effect of fire on underwater tunnels is the damage and destruction of the main structure due to high temperatures, resulting in the damage and loss of its bearing capacity and function. Furthermore, due to the unique climatic conditions of underwater tunnels, fires are often more severe than those found in overland traffic tunnels, and repairs are extremely complex [7,8,9,10]. The steel–concrete–steel composite structure has considerable benefits over typical reinforced concrete schemes, such as a high bearing capacity, good waterproof performance, minimal prefabricated site needs, fast building speed, and so on. It is better suited to large immersed tunnels and will be the future development path for large immersed tunnels. As a result, future studies on the fire mechanical response of steel–concrete–steel composite structures will play a critical role in fire safety design, tunnel construction, and operation safety of large-scale submerged tunnels.

Steel–concrete composite structural components are commonly utilized in building constructions as vertical and horizontal load-bearing components, and they are exposed to fire directly. As a result, the majority of research on the fire resistance of composite structures focuses on the construction of composite beam structures. Lennon and Moore (2003) conducted seven fire experiments on eight-story full-scale composite structures and discovered that steel–concrete composite beams could preserve stability and integrity even when deflected significantly [11]. Wang et al. (2017) studied the effect of various shear connection degrees on partially shear-connected composite beams under fire and proposed an analytical method for predicting the fire response of composite beams with steel–concrete partial shear connections, taking into account the effect of temperature on material properties as well as the slip behavior of shear connections under high temperatures [12,13]. Zhou et al. (2005) used a finite element model verified by the test results of simply supported composite beams to analyze and study the relevant parameters such as concrete slabs, steel beams, studs, loads, and fire protection layers of steel beams that affect the fire resistance of composite beams. The load ratio and the thermal resistance coefficient (or thickness) of the fire protection layer of steel beams are two critical characteristics that influence the fire resistance performance of composite beams, according to the findings [14]. Wang et al. (2015) studied the fire resistance of composite steel beams with reinforced truss floor bearing plates considering the shear slip of bolts. The research results showed that the composite steel beams with a fully shear resistance design had better fire resistance at room temperature, but shear slip and lift displacement still occurred at high temperatures [15]. Ma (2014) conducted a finite element numerical simulation analysis of the fire resistance of the honeycomb composite beam and revealed the temperature field distribution and failure mode of the honeycomb composite beam under fire [16]. Han (2020) conducted an experimental study on the fire resistance of stainless steel and concrete composite beams [17]. The test found that the composite beams all showed bending-shear failure modes under fire, and the specimens had large rebound deformation after the test. Li et al. (2010) proposed the critical temperature method for the fire resistance design of simply supported composite beams according to the method of checking the fire resistance of simply supported composite beams in the Code for Fire Safety of Steel Building Structures (GB51249-2017), and they provided the corresponding critical temperature calculation method under different floor thicknesses, fire resistance limits and load ratios [18]. Song et al. (2008) summarized some research results obtained by domestic and foreign researchers on the fire resistance of composite structures and summarized the working mechanism and fire resistance design methods of composite structures under fire, as well as some new progress in fire resistance research. For an immersed tunnel with a steel–concrete–steel composite structure, the stability and durability of the composite structure under a high fire temperature are of great significance to the safety of the whole tunnel structure, because the external steel shell has both waterproof and load-bearing functions [19]. Song et al. (2019) carried out three flexural beam tests and three shear beam tests for the steel–concrete–steel composite structure. Based on the test results, a shear design method combining the shear mechanism of steel–concrete composite with the shear mechanism of the I-steel web was proposed [20]. At present, the research foundation for the mechanical response and failure mode under a high temperature of fire, and the coordination of stress and deformation under a high temperature of the steel–concrete–steel composite structure are weak.

In this paper, a study on the mechanical behavior of the steel–concrete–steel composite structure under a high temperature of fire was carried out based on the physical model test and numerical simulation analysis for the steel–concrete–steel composite structure used in the immersed tube tunnel of the Shenzhen–Zhongshan link, revealing the temperature transfer mechanism, component damage mode, and structural mechanical response characteristics in the internal structure of the composite structure under a high temperature of fire. Related results can provide a reference for the research on fire protection technology and fire mechanical behavior of steel–concrete–steel immersed tunnels.

## 2. Structural Form of Immersed Tube Tunnel Sections

**Figure 1:**
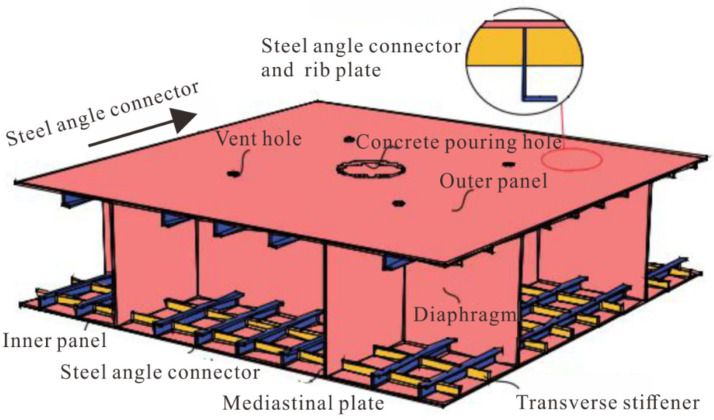
The steel–concrete–steel composite structure.

At present, the main tube structure of an immersed tunnel is mainly divided into reinforced concrete and steel plate concrete. The initial immersed tunnel adopted the form of a double steel plate or single steel plate. The outer steel plate was mainly used for waterproofing and as concrete pouring formwork, and the inner side was a reinforced concrete structure, so the outer steel plate was often not used as a load-bearing structure. With the advancement of immersed tunnel technology, in the 1980s, immersed tunnels with steel plate–concrete composite structures as the main bearing structure achieved engineering applications. In Japan, a new type of steel–concrete–steel composite structure was adopted in the Hong Kong Island Tunnel of Kobe Port: the inner structure was equipped with horizontal and vertical steel partitions, and different types of stiffeners were installed in the horizontal and vertical directions of the tunnel. The vertical and horizontal partitions and the inner and outer steel plates formed a whole to bear the external load, so that the component of the composite structure could fully utilize their respective material characteristics, as shown in Figure 1.

## 3. Experimental Scheme of Mechanical Behavior of the Steel–Concrete–Steel Composite Structure under a High Temperature of Fire

### 3.1. Processing of Components

**Figure 2:**
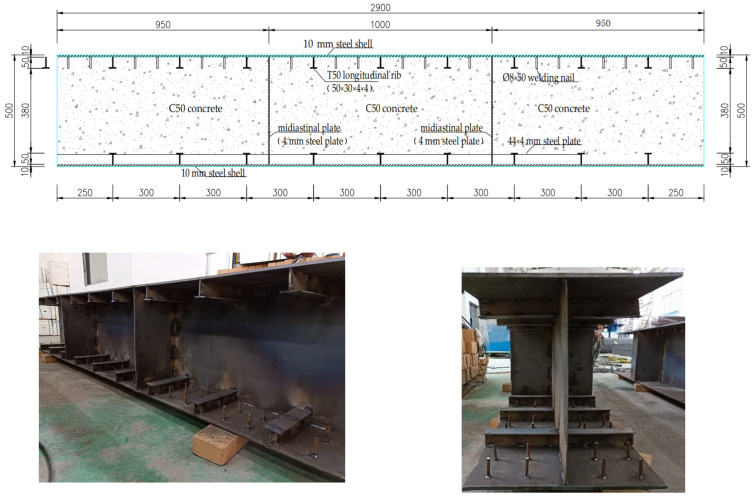
The specific construction of the steel structures.

The size of the steel–concrete–steel composite structural component was 2.9 m × 0.5 m × 0.5 m, the steel plate type was Q355B, the concrete label was C50, and there were horizontal and vertical steel plates, longitudinal T-ribs, welding studs, etc. The thickness of the steel plate at the top and bottom of the component was 10 mm, and the thickness of the transverse and longitudinal partitions was 4 mm. The specific structure of the steel structural component is shown in Figure 2.

After the steel structure was fabricated, thermocouples were arranged inside the component, the concrete was poured and cured, and the curing time was not less than 7 days.

### 3.2. Arrangement of Measuring Points

During the test, internal thermocouples were used to measure the temperature transfer law at different positions inside the component, and wire-pull displacement gauges were used to measure the vertical deformation of the steel plate at the top of the component.

#### 3.2.1. Arrangement of Internal Temperature Measuring Points

**Figure 3:**
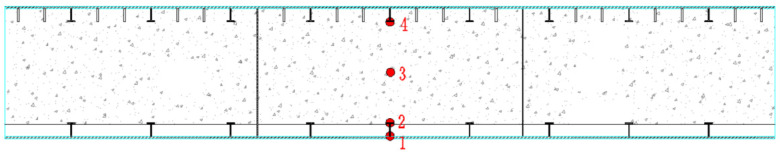
Arrangement of the measuring points of temperature in the structure.

The temperature measuring points inside the component were arranged nonuniformly along with the height of the section, and a total of four thermocouples were arranged on the measuring section. The distance between each measuring point and the upper surface of the steel plate at the bottom of the component was 0 mm (measuring point 1), 10 mm (measuring point 2), 240 mm (measuring point 3), and 470 mm (measuring point 4), respectively, as shown in Figure 3.

#### 3.2.2. Arrangement of the Measuring Points of the Roof Displacement

**Figure 4:**
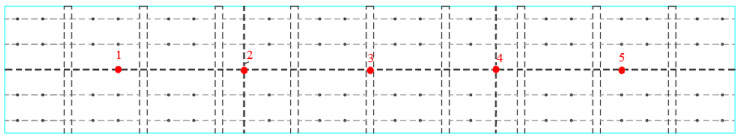
Arrangement of the measuring points of the roof displacement of the structure.

A total of five pull-wire displacement gauges were arranged on the top of the component, and the ends of the pull-wire were fixed on the top plate of the component. The specific arrangement positions were as follows: 1 in the middle of the component’s clear span (measuring point 3), 1 at each of the 1/4 component’s clear span (measuring point 2 and 4), and 1 at each end on both sides (measuring point 1 and 5), as shown in Figure 4.

### 3.3. Test Implementation

**Figure 5:**
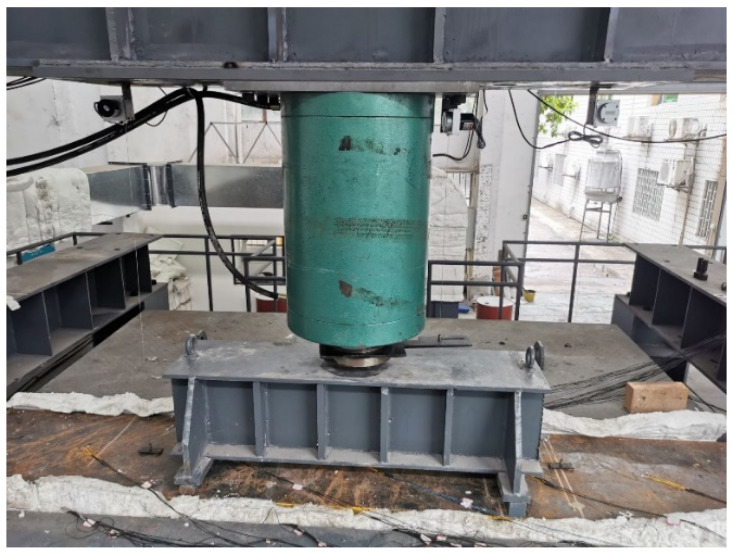
The picture of the test setup.

**Figure 6:**
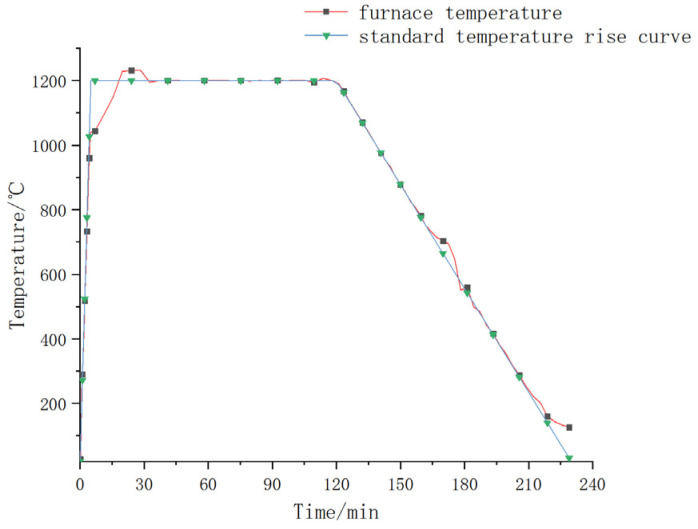
Temperature rise curve in the furnace during the test.

The experiment was carried out with the test setup as shown in Figure 5. The roof components of the immersed tunnel were selected to conduct scale model testing under the most unfavorable condition. In this experiment, the geometric similarity scale was 1/3, and the elastic modulus similarity scale was 1. According to the similarity criterion, the similarity scale of concentrated force load was 1/9. According to the engineering design data, under the most unfavorable working condition, the design concentrated force load of the prototype structure was 1440 kN; therefore, the concentrated load of this experimental model was 1440/9 = 160 kN. The steel plate at the bottom of the component was directly affected by the fire, and the RABT standard temperature rise curve was adopted. A constant concentrated load was applied to the midspan position of the steel plate at the top of the component. The mechanical response and structural damage characteristics of the steel–concrete–steel composite structure under a high temperature of fire were analyzed. The specific implementation process was as follows: (1) lift the component to a predetermined position above the furnace with a crane, and place the component so that both ends of the component are on the support of the loading reaction beam; (2) carry out fire protection for the external wiring of the thermocouples, and install the loading system of the fire test furnace; (3) install the pull-wire displacement gauges, and the test data response is normal; (4) add fireproof cotton between the side of the component and the furnace cover to ensure that the component is under fire on one side; (5) apply a predetermined load to the top steel plate and turn on the burner for the test after the data are stable. The corresponding relationship between the temperature rise curve in the furnace and the RABT standard curve during the test is shown in Figure 6.

## 4. Analysis of Test Results

### 4.1. The Overall Test Phenomena

The damaged state of the component after the test is shown in Figure 6. It can be seen from the figure that due to the influence of the high temperature, the color of the concrete near the bottom steel plate has changed significantly, from blue-gray to gray-white. The color change area is about 10 cm in height, and the closer it is to the bottom steel plate, the more broken the concrete is; the concrete is powdery within 3 cm from the upper surface of the component bottom plate.

**Figure 7:**
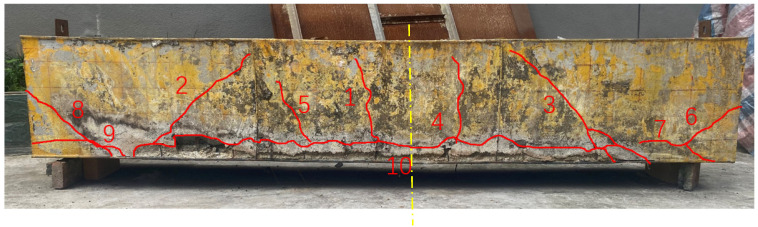
Distribution of cracks of the structure after test.

Due to the influence of the high temperature, the crack development on the side of the component is obvious, and the main distribution positions of the cracks are shown in Figure 7. It can be seen from the figure that under the action of thermal–mechanical coupling, the cracks couples, such as crack 1 and crack 4, crack 2 and crack 3, crack 6 and crack 8 distribute symmetrically according to the midspan line (dash-dot line in Figure 7). A shear failure model can be observed in the appearance of the specimen after the fire. An internal structure of the component comparison found that the side cracks of the component were mainly concentrated in the vicinity of the partition plates and the T-ribs. The crack statistics are shown in Table 1. This failure is mainly caused by the temperature internal force caused by the temperature gradient and thermal expansion difference in the component. When affected by the high temperature of the fire, a large temperature difference occurs at the interface between the steel plate and the concrete, resulting in a local thermal stress concentration, and then damage cracks. At the same time, the expansion and penetration of cracks are aggravated under the action of the external load.

### 4.2. Analysis of Temperature Transfer and Deformation Law of Composite Structures under a High Temperature of Fire

#### 4.2.1. Temperature Distribution Law in Component

**Figure 8:**
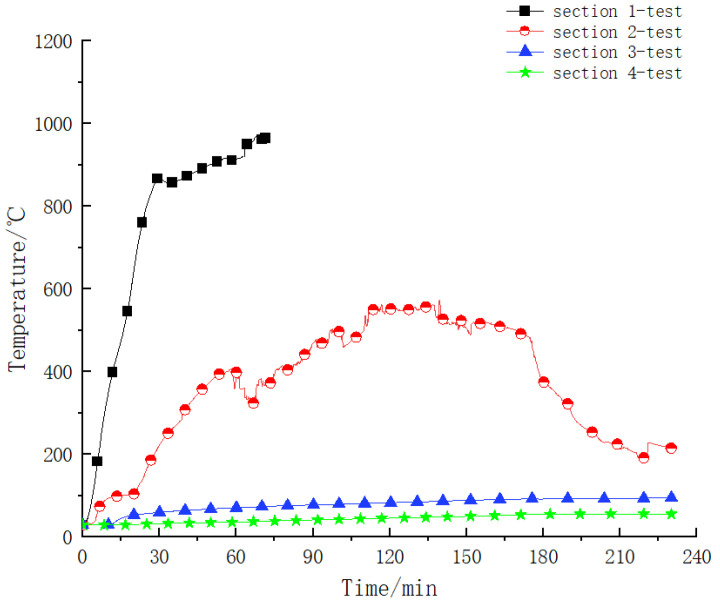
Temperature versus time curve for different sections during the test.

Firstly, the temperature distribution law in the steel–concrete–steel composite structure under the RABT heating curve was systematically analyzed. The temperature versus time curve for different sections in the component is shown in Figure 8. It can be seen from the figure that Section 1 (0 mm from the bottom plate) was directly affected by the high temperature of the fire during the test process. The temperature change law of this monitoring point was similar to the temperature rise curve in the furnace, and there were obvious heating and cooling stages: within the first 30 min, the temperature of the monitoring point was in the stage of rapid heating, and the maximum temperature reached 1007 °C; at 30~60 min, the temperature in the furnace was stable at 1200 °C, and the temperature of the monitoring point was stable at about 1050 °C, which was slightly less than 1200 °C, indicating that although the measuring point was directly affected by the high temperature, the temperature transfer in the component had a certain hysteresis; after 60 min, the temperature of the measuring point was abnormal, and the reason may have been that the range of the thermocouple embedded in the component was 800~1000 °C. After the thermocouple was continuously affected by the high temperature, the performance of the thermocouple was compromised, resulting in subsequent abnormalities in the temperature acquisition data.

During the test, the temperature change rule of Section 2 (10 mm from the bottom plate) was similar to Section 1, but due to the hysteresis of the temperature transfer, in the initial heating stage, the temperature rise rate of Section 2 was significantly lower than that of Section 1, and the temperature reached the maximum value of 714 °C at 140 min; in the 20 min furnace cooling, the temperature of Section 2 entered the cooling stage, and the temperature of Section 2 was 321 °C when the test was completed.

The temperature of Section 3 (240 mm from the bottom plate) and Section 4 (470 mm from the bottom plate) was only in the growth stage, and there was no obvious decline stage, indicating that the hysteresis effect of the internal temperature transfer of the component was very obvious, with the highest temperature in Section 3 being 135 °C and the highest temperature in Section 4 being 61 °C.

#### 4.2.2. Variation Rule of Roof Displacement

**Figure 9:**
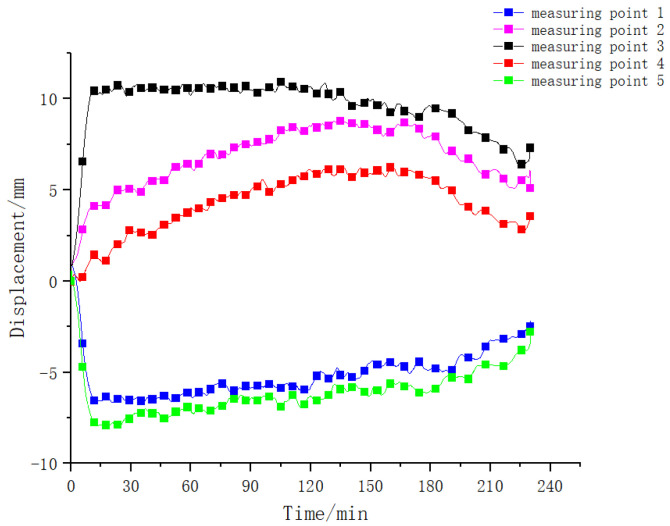
Displacement versus time curve of different monitoring points during the test.

The variation law of the displacement of different measuring points on the top plate is shown in Figure 9. It can be seen from the figure that the variation law of the displacement of different measuring points during the test is very obvious. The three measuring points (measuring points 2, 3, and 4) located on the clear span of the component produced a vertical downward deformation; the measuring points (measuring points 1 and 5) at the end of the component produced a vertical upward deformation, indicating that under the effect of thermal–mechanical coupling, the overall deformation trend was that the middle sinks and the two ends rise. Moreover, the deformation law of the measuring points during the test was affected by the high temperature of the fire, showing three obvious stages: (1) in the rapid growth stage, at the beginning of the test, affected by the rapid heating stage in the furnace, the deformation of the component increased rapidly. The midspan position reached the maximum value of 11 mm; (2) in the stable deformation stage, the temperature in the furnace was stable at 1200 °C, and the deformation of the component was less affected by the temperature and remained basically stable; (3) in the deformation recovery stage, affected by the cooling stage in the furnace, the temperature in the component decreased, the deformation of the component was partially restored, and the residual deformation was 7 mm. This phenomenon showed that the deformation law of the steel–concrete–steel composite structure was closely related to its fire boundary conditions under the action of the thermal–mechanical coupling, and the effect of a high fire temperature on the mechanical mechanism of the components was very obvious.

## 5. Numerical Simulation Research on Mechanical Behavior of Steel–Concrete–Steel Composite Structure

In order to further analyze the mechanical behavior of the steel–concrete–steel composite structure under the action of the thermal–mechanical coupling, a comparative study was carried out between the numerical simulation and the physical model test results.

### 5.1. Material Parameters

#### 5.1.1. Thermal Parameters

The thermal parameters of steel and concrete are shown in Table 2. The thermal parameters of ordinary concrete at high temperatures should be determined according to the following regulations.

The heat transfer coefficient of concrete $\lambda_{c}$ should be calculated according to Equation (1):

(1) $$\lambda_{c}=1.68-0.19\frac{T_{c}}{100}+0.0082{\left(\frac{T_{c}}{100}\right)}^{2}$$

The specific heat capacity of concrete $c_{c}$ should be calculated according to Equation (2):

(2) $$C_{c}=890+56.2\frac{T_{c}}{100}-3.4{\left(\frac{T_{c}}{100}\right)}^{2}$$

where: *T_c_*—the temperature of the concrete (°C);

$\lambda_{c}$—the heat transfer coefficient of concrete (W/(m·°C));

$c_{c}$—specific heat capacity of concrete (J/(kg·°C)).

Considering the influence of moisture on the concrete’s temperature, the main influence is the change of the concrete’s specific heat capacity, so the concrete’s specific heat capacity is corrected. Assuming that the vaporization of all water is completed between 90 °C and 110 °C, considering that the general concrete moisture content is 4%, and the vaporization heat of water (at 100 °C, one-atmosphere pressure) is 2257.2 kJ/kg, the density of concrete is taken as 2300 kg/m^3^. The heat required for water vaporization in one cubic meter of concrete is:

(3) 2300×0.04×2257.2=207,662.4(kJ)

The specific heat of concrete at 100 °C is 942.8 J/(kg·°C) according to Equation (2), so the heat required for one cubic meter of concrete to heat up to 20 °C is:

(4) 2300×20×942.8=43,368.8(J)

Therefore, the specific heat value of concrete between 90 °C and 110 °C is:

(5) $$\frac{207,662,400+43,368.8}{2300\times 20}=5103.2(kJ/(\mathrm{kg}\;{}^{\circ}C))$$

The specific heat of concrete in other temperature sections was taken according to Equation (2).

#### 5.1.2. Heat Transfer Parameters

There are three basic ways of transferring heat: heat conduction, heat convection, and heat radiation. In this model, the heat transfer modes of the flue gas in the overfire area were thermal convection and thermal radiation.

The comprehensive emissivity was taken from the American standard ANSI/AISC360-10 “Specification for Structural Steel Buildings” (2010), which comprehensively considers the emissivity of the flue gas and the influence of the radiation angle coefficient [21]. The comprehensive emissivity of the selected steel shell concrete surface was taken as 0.7, and the heat dissipation coefficient of the film layer was taken as 25.0 w/m^2^/°C.

#### 5.1.3. Material Constitutive Relations at High Temperature

The tensile behavior of the concrete is linear elastic until the tensile strength. The stress–strain relationship of the compressive part of the high-temperature concrete adopts the high-temperature constitutive relationship proposed by Lie, as shown in Equation (6) [22].

(6) $$\sigma_{c,T}=\left\{ \begin{array}{l} f_{c,T}[1-{\left(\frac{\varepsilon_{cu,T}-\varepsilon_{c,T}}{\varepsilon_{cu,T}}\right)}^{2}]\begin{array}{cccc} & & & \varepsilon_{c,T}\leq \varepsilon_{cu,T} \end{array}\\ f_{c,T}[1-{\left(\frac{\varepsilon_{cu,T}-\varepsilon_{c,T}}{3\varepsilon_{cu,T}}\right)}^{2}]\begin{array}{cccc} & & & \varepsilon_{c,T}>\varepsilon_{cu,T} \end{array} \end{array}\right.\;\varepsilon_{cu,T}=0.0025+(6T+0.04T^{2})\times 10^{-6}\;f_{c,T}=\left\{ \begin{array}{ll} f_{c,20} & \varepsilon_{c,T}\leq \varepsilon_{cu,T}\\ f_{c,20}[2.011-\frac{2.353(T-20)}{1000}] & \varepsilon_{c,T}>\varepsilon_{cu,T} \end{array}\right.$$

where: *σ_c,T_*—stress of concrete at high temperature;

*f_c,T_*—compressive strength of concrete at high temperature;

*ε_c,T_*—strain of concrete at high temperature;

*ε_cu,T_*—strain of concrete corresponding to peak stress at high temperature

*T*—temperature.

The reduction coefficient of the concrete’s tensile strength at high temperature was set according to the provisions of European specification EC2, as shown in Equation (7) [23].

(7) $$K_{c,T}=\{\begin{array}{l} 1.0\;\;\;\;\;\;\;\;\;\;\;\;T\leq 100{}^{\circ}C\\ 1.0-1.0\times \frac{T-100}{500}\;\;\;\;100{}^{\circ}C<T\leq 600{}^{\circ}C \end{array}$$

where *K_c,T_*—the reduction coefficient of concrete tensile strength at high temperature.

In the analysis, the constitutive model of the steel at different temperatures adopted the ideal elastic–plastic model. The yield strength of steel at room temperature was 355 MPa, and that of the elastic model was 2.06 × 10^5^ N/mm^2^. The elastic modulus and yield strength reduction coefficient at high temperatures were taken according to the provisions of the Code for Fire Safety of Steel Building Structures GB51249, as shown in Equation (8) [24]. The temperature has little effect on the Poisson’s ratio of steel, and the Poisson’s ratio takes a constant value of 0.3.

(8) $$\chi_{T}=\{\begin{array}{l} \frac{7T_{s}-4780}{6T_{s}-4760}\;\;20{}^{\circ}C\leq T_{s}<600{}^{\circ}C\\ \frac{1000-T_{s}}{6T_{s}-2800}\;\;600{}^{\circ}C\leq T_{s}<1000{}^{\circ}C \end{array}\;\eta_{T}=\{\begin{array}{l} 1.0\;\;\;\;\;\;\;\;\;\;\;\;20{}^{\circ}C\leq T_{s}<600{}^{\circ}C\\ 1.24\times 10^{-8}{T_{s}}^{3}-2.096\times 10^{-5}{T_{s}}^{2}\\ +9.228\times 10^{-3}T_{s}-0.2168\;\;\;300{}^{\circ}C\leq T_{s}<800{}^{\circ}C\\ 0.5-T_{s}/2000\;\;\;\;\;\;\;\;800{}^{\circ}C\leq T_{s}<1000{}^{\circ}C \end{array}$$

where: *χ_T_*—the reduction coefficient of strength of steel at high temperature;

*η_T_*—the reduction coefficient of Young’s modulus of steel at high temperature.

### 5.2. Finite Element Model

**Figure 10:**
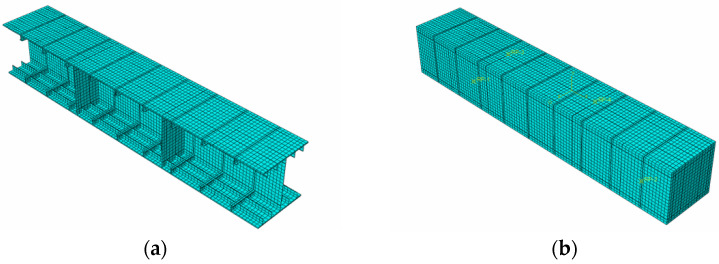
The overall numerical model. (**a**) Model for the steel structure, (**b**) model for the concrete structure.

**Figure 11:**
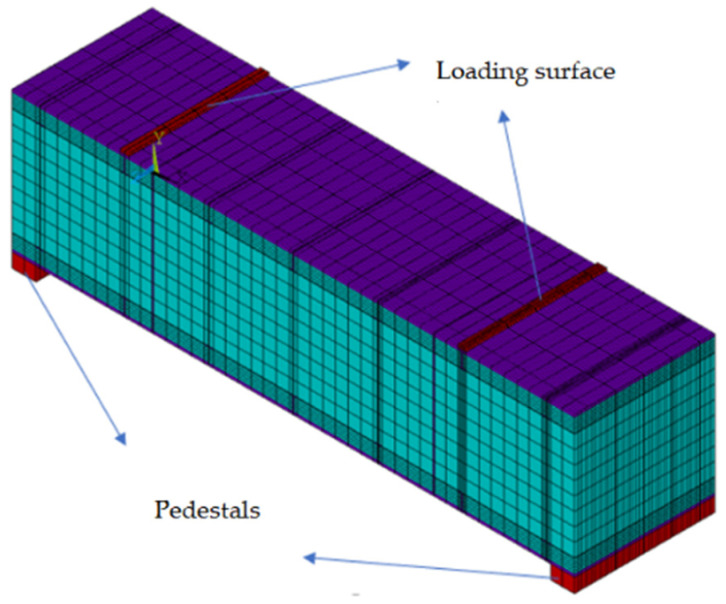
Schematic diagram of the loading mode of the finite element model.

There are many factors affecting the distribution of structural temperature fields under fire. In order to simplify the analysis process, the following assumptions were considered:

1. Steel and concrete are homogeneous and isotropic structural materials, and their thermal parameters such as thermal conductivity and specific heat capacity are the same in each direction at the same temperature;
2. A complete heat transfer between steel and concrete, ignoring the influence of contact thermal resistance;
3. The influence of structural high-temperature mechanical response on structure temperature field distribution is not considered;

The sequential thermal–mechanical coupling method was used to analyze the temperature field of the structure first, and then the results of the temperature analysis were applied to the mechanical model as loads to analyze the mechanical response of the model. The established finite element model is shown in Figure 10. The schematic diagram of the loading mode of the finite element model is shown in Figure 11. The load and the constraint configuration of the numerical model was consistent with the model test. As shown in Figure 11, using the loading surface to simulate the role of the distribution beam in the test, both ends of the model were placed on the pedestals and the displacements at the pedestals were constrained.

### 5.3. Comparative Analysis of Physical Model Test and Numerical Simulation Results

(1) Comparative analysis of temperature transfer laws

**Figure 12:**
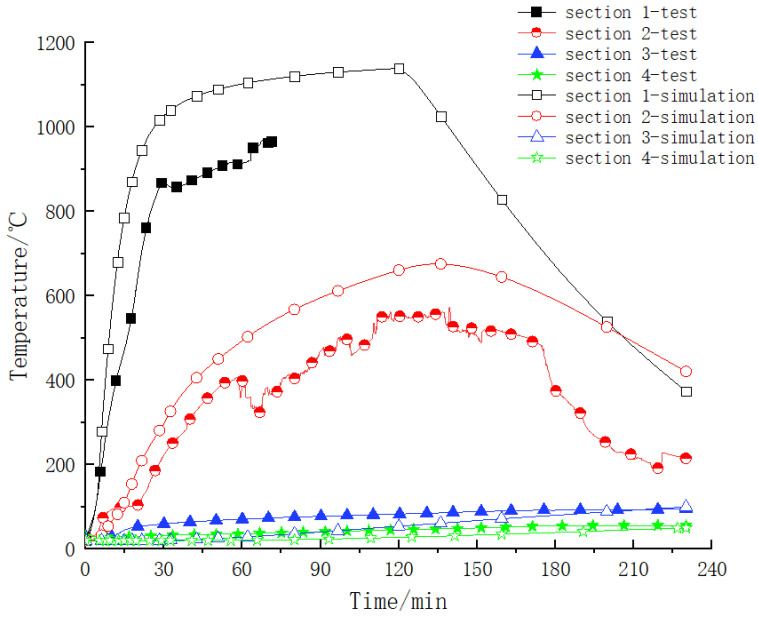
Comparative analysis of temperature transfer law of measuring points for each section.

The comparison results of the temperature–time curves are shown in Figure 12. It can be clearly seen from the figure that the numerical results are in good agreement with the physical test data as a whole, and the temperature distribution law of each section is obviously terraced. The larger the distance between the measurement points and the bottom steel plate, the lower the temperature. Due to the hysteresis effect of the temperature transfer, the heating period is longer. For Section 1 and Section 2, which are closer to the fire surface, the numerical simulation results at the same time are slightly larger than the experimental data. The possible reasons are as follows: there is a certain difference between the simulation of boundary conditions and the actual situation; there are positional deviations due to pouring and other reasons during the production of the component; and the thermal parameters of the material are different from the actual ones.

(2) Contrastive analysis of roof deformation laws

**Figure 13:**
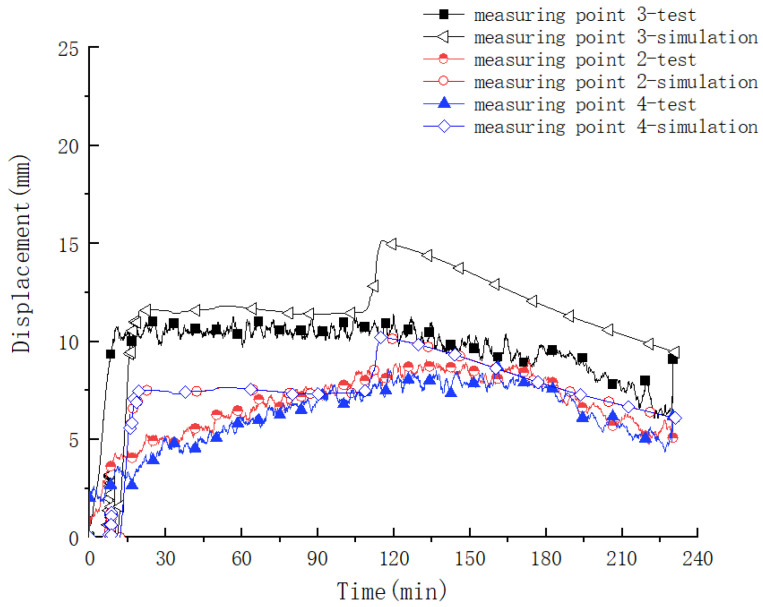
Comparative analysis of deformation development law of measuring points of clear span area of composite structure.

The comparison results of the roof deformation laws are shown in Figure 13. It can be seen from the figures that the displacement obtained by numerical simulation and the displacement measured by the test are basically the same when the component is exposed to fire. Within half an hour before the fire, the difference between the two is very small, then there is a certain deviation where the displacement obtained by numerical simulation is larger than that measured by experiment. The overall displacement change has three stages: (1) A rapid growth stage: within 15 min of heating, the displacement rises faster. The main reason is that the temperature gradient of the section is large at this time, and the bending deformation of the section due to uneven thermal expansion increases rapidly. (2) A deformation stability stage: at this time, although the average temperature of the section increases, the temperature gradient of the section decreases. The displacement of the section increases due to the decrease of stiffness caused by the high temperature, but the decrease in temperature gradient reduces the bending caused by an uneven thermal expansion, and the overall displacement remains stable, or slightly increases. (3) A deformation recovery stage: after the temperature in the furnace decreases, the stiffness of the component is recovered somewhat, the temperature gradient in the section further decreases, and the displacement partially recovers, but the recovery speed is slow. The possible reason for the deviation is that in the numerical simulation, due to the large internal force of the temperature, the cracks in the concrete have a significant impact on the stiffness of the beam, and the deformation increases.

(3) Distribution analysis of structural plastic zone under a high temperature of fire

**Figure 14:**
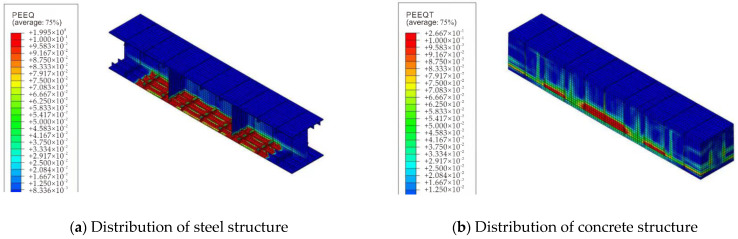
Distribution of the plastic zone of the composite structure under fire.

Based on the numerical simulation results, the plastic zone distribution of the steel–concrete–steel composite structure under a high temperature of fire was obtained, as shown in Figure 14. It can be seen from the figure that the lower flange is close to the fire surface and the plastic strain at the lower flange of the component is relatively large, mainly due to the fact that the higher the temperature, the greater the degradation of the material strength and elastic modulus; the thermal expansion deformation is constrained by the lower temperature concrete above, and the material enters a plastic state. The distribution of the plastic zone in other parts of the component is less, indicating that the high temperature of fire has a limited influence on the plastic development of the entire structure.

(4) Analysis of structural crack distribution under a high temperature of fire

**Figure 15:**
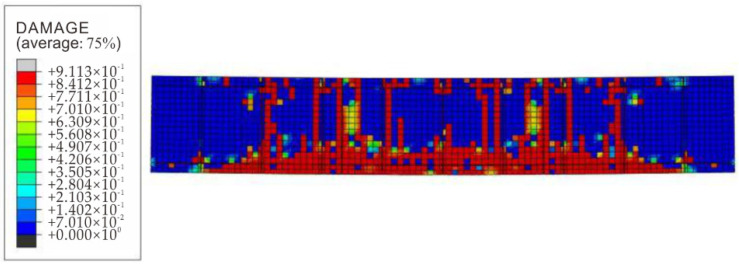
Distribution of failure of concrete in the composite structure caused by a high temperature under fire.

Based on the numerical simulation results, the tensile failure cloud diagram of the component concrete was obtained, as shown in Figure 15, which was consistent with the physical model test results: the overall component crack distribution had a good symmetry. There was a longitudinal crack running through the component in parallel to the lower flange, which was consistent with the position of the test no. 10 crack. There was a 45° inclined crack on the connection line between the beam end support and the loading point. The midspan crack developed vertically, and the crack distribution was in good agreement with the test results. It shows that the numerical simulation can well simulate the failure of the component concrete under a high temperature of fire.

## 6. Conclusions

(1) Under the RABT standard temperature rise curve, the area directly affected by the high temperature under fire in the steel–concrete–steel composite structure was within a range of about 10 cm from the fire surface, and within 3 cm away from the fire surface, the concrete showed a powdered state; the crack distribution modes in the composite structures under thermal–mechanical coupling were given.
(2) The temperature transfer law of the measuring points at different depths of the combined structure were given: the temperature distribution of the measuring points closer to the fire surface presented a stepped distribution, and there was an obvious heating and cooling stage; the temperature of the far measuring points showed a continuous growth law due to the hysteresis effect of temperature transfer; the results of the numerical simulation and physical model test were compared to verify the temperature transfer law in the composite structure under a high temperature of fire.
(3) The evolution law of the roof displacement of the composite structure was given: the overall deformation trend of the structural component under the action of thermal–mechanical coupling was that the middle sank and the two ends upturned. Affected by the high temperature of the fire, the roof deformation presented three distinct stages with time: a rapid growth stage, a deformation stabilization stage, and a deformation recovery stage. The results of the numerical simulation and physical model test were compared to verify the mechanical response mechanism of composite structure under the high temperature of fire.
(4) The high temperature of fire had limited impact on the plastic development of the whole structure. The plastic zone was concentrated in the lower flange near the fire surface, and the plastic zone of the other parts was less distributed.
(5) The composite structure presented a shear failure mode as a whole: the crack distribution of the whole component had good symmetry, a 45° oblique crack appeared on the line connecting the beam end support and the loading point, and the midspan cracks developed vertically.

## Figures and Tables

**Table 1:** Statistics of cracks of the structure after test.

Crack Number	Crack Type	Development Direction	Crack Length (cm)
1	Vertical crack	Vertical	34
2	Oblique crack	Slanted 45° inward	57
3	Oblique crack	Slanted 45° inward	57
4	Vertical crack	Vertical	34
5	Vertical crack	Vertical	34
6	Oblique crack	Obliquely outward	17
7	Oblique crack	Obliquely outward	9
8	Oblique crack	Obliquely outward	21
9	Oblique crack	Obliquely outward	11
10	Horizontal crack	Longitudinal along the component	210

**Table 2:** The thermal parameters for materials.

Material	Density kg/m^3^	Heat Transfer Coefficient w/m/°C	Specific Heat Capacity J/m/°C
Steel	7850	45.0	600
Concrete	2300	With the change of temperature, Equation (1)	With the change of temperature, Equation (2)

## Data Availability

Data available on request.
